# T cell receptor excision circles as a tool for evaluating thymic function in young children

**DOI:** 10.1590/1414-431X20198292

**Published:** 2019-06-19

**Authors:** A. Levy, A. Rangel-Santos, L.C. Torres, G. Silveira-Abreu, F. Agena, M. Carneiro-Sampaio

**Affiliations:** 1 Instituto da Criança, Hospital das Clínicas, Faculdade de Medicina, Universidade de São Paulo, São Paulo, SP, Brasil; 2 Laboratório de Pesquisa Translacional, Instituto de Medicina Integral Prof. Fernando Figueira (IMIP), Recife, PE, Brasil; 3 Instituto Central, Hospital das Clínicas, Faculdade de Medicina, Universidade de São Paulo, São Paulo, SP, Brasil

**Keywords:** Human thymus, TRECs, Thymic function, Infants

## Abstract

The thymus is a primary lymphoid organ responsible for the maturation of T cells as well as the immunological central tolerance. It is in the antenatal period and infancy that it plays its major role. In clinical practice, T cell receptor excision circles (TRECs) are considered a direct and reliable measure of the thymic function. TRECs are a by-product of DNA formation in gene rearrangement of T cell receptors. They are stable and they do not duplicate during mitosis, representing the recent emigrant T cells from the thymus. Despite their importance, TRECs have been neglected by physicians and there is a lack of data regarding thymic function during infancy of healthy children. In order to evaluate thymic function in the first years of life, we propose measuring TRECs as a valuable tool. One hundred and three blood samples from children and adolescents between 3 months and 20 years of age were analyzed. The mean TRECs count was 136.77±96.7 copies of TRECs/μL of DNA. The individuals between 0 and 5 years of age had significantly higher TRECs values than those between 10 and 20 years of age. No significant difference was observed in TRECs values among age groups below 5 years of age. An inverse correlation between TRECs and age was found (r=0.3 P=0.003). These data highlight and validate the evidence of decreased thymus function with age, even during infancy. Awareness should be raised with this important albeit ignored organ.

## Introduction

The thymus is a primary lymphoid organ responsible for the maturation of T cells as well as immunological central tolerance. Despite its relevance in early immunity, the thymus has been neglected by physicians (1). Unlike bone marrow function, which is the counterpart to thymic function in relation to B cells, thymic function is rarely evaluated in everyday medical practice. A number of ignored aggressions to the thymus occur, such as the use of corticosteroids and other immunosuppressive drugs, systemic infections, and even hormonal changes (2).

To assess these mentioned aggressions, an effective way of measuring thymic function is needed. Currently, thymic function can be indirectly measured by computerized tomography imaging, PET scans, and flow cytometry for T cell subpopulations (3). More recently, at the beginning of this century, a direct measurement method with T cell receptor excision circles (TRECs) was developed (4). TRECs are a byproduct of DNA formation during gene rearrangement of T cell receptors. Because TRECs are stable and do not duplicate during mitosis, TRECs in the peripheral blood represent a valid biomarker of recent thymic function; thus, TREC-positive cells are called recent thymic emigrants. For a few conditions, evaluations to measure TRECs are usually conducted in clinical practice (5): i) newborn screening tests for severe combined immunodeficiency (SCID), ii) evaluation of immune reconstitution during antiretroviral therapy in AIDS patients, and iii) testing T cell reconstitution after bone marrow transplantation. TRECs counts have also been used to evaluate thymic function in SCID and DiGeorge patients (5). In addition to these relevant uses, TRECs counts can be useful for evaluating thymic function, particularly in early life, which is considered the period when the role of the thymus is most important (6). To the best of our knowledge, normal values of TRECs in early age have not been deeply explored. Some authors have previously studied TRECs in normal populations; however, these studies included few individuals in their first years of life (6,7).

In the present paper, we proposed to study TRECs in healthy children and adolescents ranging between 0-20 years of age.

## Material and Methods

This study was conducted at the Instituto da Criança, Hospital das Clínicas, Faculdade de Medicina da Universidade de São Paulo, a tertiary university hospital in São Paulo, from January 2016 to August 2018. One hundred and three healthy children and adolescents between 3 months and 20 years of age were selected for this study. All of the individuals were recruited during routine pediatric outpatient visits or during preoperative evaluation for minor surgical procedures, such as posthectomy or inguinal hernia correction. The exclusion criteria were presence of any chronic or acute systemic disease, past medical history of any significant disease, or corticosteroid use in the previous month. All parents or representatives provided informed consent for the use of a small blood sample for this study, whose protocol was approved by the HC-FMUSP Ethics Committee (CAPPesq number 335.543). Blood was drawn for other kinds of analyses requested by the attending pediatrician or pediatric surgeon.

Peripheral venous blood samples (2-5 mL) were collected using EDTA Vacutainer blood collection tubes (Becton-Dickinson, Brazil). Genomic DNA was extracted from peripheral blood with Qiagen columns (QIAamp DNA minikit; Germany) according to the manufacturer's instructions. The DNA concentration and purity were determined using a NanoVue Plus spectrophotometer (GE Healthcare, UK). TRECs concentrations were analyzed by real-time quantitative PCR (StepOne PlusTM, Applied Biosystems, USA) using TaqMan Gene Expression Master Mix (Applied Biosystems). The RT-qPCR reactions were performed in a final volume of 25 μL containing 20 μM TREC primer or ACTB primer, 15 μM 6FAM labeled TAMRA TREC, and 6VIC-labeled TAMRA ACTB probes (all from Integrated DNA Technology, USA). In each reaction, the DNA sample was tested in triplicate. The measurement of ACTB signal was performed when the TRECs level of a sample was low (<25 copies/μL of DNA) (8). The reactions were carried out with an initial cycle at 50°C for 2 min and a heating cycle at 95°C for 10 min, followed by 40 cycles of 30 s at 95°C, and 1 min at 60°C. A standard curve was included in every PCR reaction for the absolute quantification of the number of TRECs per μL of DNA in each sample. The TREC standard curve was established using seven 10-fold serial dilutions that ranged from 102 to 106 TREC copies/μL of plasmids containing a TREC fragment. All analyzed RT-qPCR assays fulfilled the quality requirements of similar slopes and R^2^ values >0.96.

### Statistical analysis

Statistical analyses were performed using the statistical package GraphPad Prism version 5.0 (USA). To investigate the normal distribution of TREC counts, the Shapiro-Wilk test was used. The Mann-Whitney test was used to compare quantitative variables between age groups, and Spearman's correlation coefficient was used to correlate quantitative variables. A P-value of less than 0.05 was considered statistically significant in all analyses.

## Results

**Figure 1.**
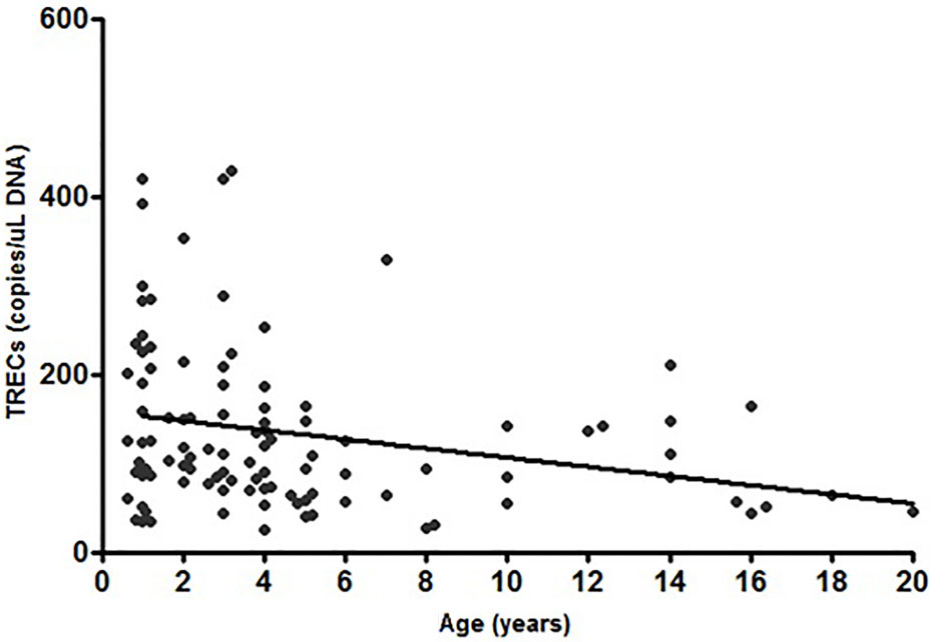
Levels of T cell receptor excision circles (TRECs) per μL of DNA in healthy children and adolescents from 3 months to 20 years of age. Spearman's correlation coefficient: rs=–0.302, P=0.0019.

The mean age of the sample was 4.98±4.63 (range 0–20 years). The mean total TREC count was 136.77±96.7 copies/μL of DNA. The individuals were divided into 7 age groups (Table 1). TREC content of our sample did not display a normal distribution. Statistically significant differences were found between the age groups of 3–12 months, 25–36 months, and 37–48 months when compared to the 121–240 months age group. The Spearman correlation test revealed a significant inverse correlation between TREC values and age (rs=–0.302, P=0.0019) (Figure 1).

**Table 1.** Levels of T cell receptor excision circles (TRECs) according to age groups.

Age (months)	No. of individuals	Copies of TRECs/μL of DNA [median (max/min)]
03–12	10	163 (45/299)
13–24	18	125 (34/430)
25–36	11	118 (79/353)
37–48	15	116 (44/420)
49–60	17	119 (52/253)
61–120	18	77 (27/330)
121–240	14	74 (44/210)

## Discussion

The main contribution of the present study was the investigation of TRECs values in a significant number of healthy young individuals from 0–5 years of age. This is currently the largest collection of TRECs data in this age group, which is when the thymus plays a major role. As expected from the experimental data, the TRECs values at an early age were significantly higher when compared to that in the second decade of life, suggesting that intrathymic proliferation decreased with age, i.e., the involution of this organ can occur even during childhood and certainly before adolescence (9).

Interestingly, young children have not only a proportionally large thymus (10) but also large secondary lymphoid organs, including mucosal-associated organs, and simultaneous peripheral blood lymphocytosis (11,12). Thus far, this phenomenon has not been completely understood. In parallel to the well-known large thymus in early life, our data suggest that thymic function is also increased during this period.

It has previously been shown that males have reduced thymic function, but we were unable to confirm this observation since our sample population comprised mostly male subjects. There is also evidence that preterm and low-birth-weight babies have lower TRECs levels at birth, but this variable was not controlled for in our study; our youngest baby was 3 months old. Other factors that could affect TREC values in healthy individuals, such as ethnic background, oxidative stress, and exposure to pollution, could not be controlled for.

The thymus is not evaluated on a daily basis by clinicians, which is mainly because the thymus is anatomically difficult to access for histopathological studies, but also due to the information gap regarding its function. We expect that this research will highlight the relevance of the thymus in the first years of life and stimulate physicians, particularly pediatricians, to be more aware about the function of this organ. Since TRECs measurements are an easy and low-cost way of evaluating thymic function, physicians should familiarize themselves with and more commonly perform day-to-day analyses of the thymus. We hope our data may contribute to this goal by stimulating interest and raising awareness of possible thymic aggressions in daily clinical practice.
